# Expired Patents

**Published:** 1907-02

**Authors:** 


### EXPIRED PATENTS.

December 24, 1906.

418108. Dental fissure drill. A. W. Browne, Prince's Bay, N. Y.

December 31, 1906.

418380. Method of preparing porcelain teeth. A. H. Parker and A. H. Stoddard, Boston, Mass.
418492. Dental plugger. W. G. A. Bonwill, Philadelphia, Pa.
418662. Process of electroplating dental plates. J. G. Ward, Newark, N. J.

January 7, 1907.

418901. Dental engine. E. T. Starr, Philadelphia, Pa.

January 14, 1907.

419299. Dental chair. B. M. Wilkerson, Baltimore, Md.
419381. Pneumatic dental plugger. F. E. Thomas and R. P. Lennox, Cambridge, England.

The above lists of patents are furnished by Davis & Davis, Solicitors of American and Foreign Patents, Washington, D. C., and St. Paul Building, New York City.

